# Sustainability of knowledge implementation in a low- and middle- income context: Experiences from a facilitation project in Vietnam targeting maternal and neonatal health

**DOI:** 10.1371/journal.pone.0182626

**Published:** 2017-08-14

**Authors:** Leif Eriksson, Anna Bergström, Dinh Thi Phuong Hoa, Nguyen Thu Nga, Ann Catrine Eldh

**Affiliations:** 1 International Maternal and Child Health (IMCH), Department of Women’s and Children’s Health, Uppsala University, Uppsala, Sweden; 2 Departmant of Public Health and Caring Sciences, Uppsala University, Uppsala, Sweden; 3 Institute for Global Health, London, United Kingdom; 4 Hanoi School of Public Health, Hanoi, Vietnam; 5 Research Institute for Child Health, Hanoi, Vietnam; 6 School of Education, Health and Social Studies, Dalarna University, Falun, Sweden; University of Liverpool, UNITED KINGDOM

## Abstract

**Background:**

In a previous trial in Vietnam, a facilitation strategy to secure evidence-based practice in primary care resulted in reduced neonatal mortality over a period of three years. While little is known as to what ensures sustainability in the implementation of community-based strategies, the aim of this study was to investigate factors promoting or hindering implementation, and sustainability of knowledge implementation strategies, by means of the former Neonatal Knowledge Into Practice (NeoKIP) trial.

**Methods:**

In 2014 we targeted all levels in the Vietnamese healthcare system: six individual interviews with representatives at national, provincial and district levels, and six focus group discussions with representatives at the commune level. The interviews were transcribed verbatim, translated to English, and analysed using inductive and deductive thematic analysis.

**Results:**

To achieve successful implementation and sustained effect of community-based knowledge implementation strategies, engagement of leaders and key stakeholders at all levels of the healthcare system is vital–prior to, during and after a project. Implementation and sustainability require thorough needs assessment, tailoring of the intervention, and consideration of how to attain and manage funds. The NeoKIP trial was characterised by a high degree of engagement at the primary healthcare system level. Further, three years post trial, maternal and neonatal care was still high on the agenda for healthcare workers and leaders, even though primary aspects such as stakeholder engagement at all levels, and funding had been incomplete or lacking.

**Conclusions:**

The current study illustrates factors to support successful implementation and sustain effects of community-based strategies in projects in low- and middle-income settings; some but not all factors were represented during the post-NeoKIP era. Most importantly, trials in this and similar contexts require deliberate management throughout and beyond the project lifetime, and engagement of key stakeholders, in order to promote and sustain knowledge implementation.

## Introduction

Recent decades have witnessed major improvements in child survival, but the present annual rate of 2.8 million neonatal deaths (i.e. deaths occurring during the first 28 days) remains a challenge [1]. Although there are effective interventions that have been estimated to avert three out of four neonatal deaths [2, 3], implementation of these interventions into practice is pending [4]. Altogether, an increased understanding is needed with regards to which knowledge implementation strategies are effective (or not), and what supports sustainability in knowledge implementation, in short- and long-term perspectives.

Several strategies are available to support knowledge implementation, such as education, decision support, organisational and patient-oriented strategies and social interaction [5]. Yet, when to apply particular strategies [5–8] and whether selected strategies are best used as single-component or multifaceted strategies [9, 10], remains unsettled. Further, studies in low- and middle-income countries focusing on knowledge implementation strategies for improving women’s and children’s health are still limited [11].

So far, it has been suggested that improving maternal and neonatal health requires community involvement and community interaction is a way forward in knowledge implementation [12, 13]. Facilitation is deemed to be a promising route for knowledge implementation, incorporating the strategy that a person with good interpersonal and group management skills assists teams of stakeholders to identify and target problems [14, 15]. Variations of this social interaction strategy have been implemented and evaluated in several low- and middle-income countries [16–20].

The NeoKIP trial (Neonatal Knowledge Into Practice, ISRCTN44599712) evaluated a three-year, low-cost community-based intervention in the Quang Ninh province in Vietnam [21]. In this trial, local stakeholder groups worked with problem solving regarding maternal and neonatal health, supported by a lay facilitator. All facilitators had received a 2-week training program on supporting groups to assess local context, prioritize perinatal issues and identify actions targeting these problems. During the intervention period (2008–11), the facilitators assisted the local stakeholder groups assembled in the 44 intervention communes. The groups included key representatives from local primary care, the village health construct, and political and non-governmental organizations. No such intervention was performed in the 46 control communes. The facilitation strategy led to a reduction of neonatal mortality by almost 50% [22]. The reduction was detected during the third intervention year, indicating that facilitation is a time-consuming process, requiring substantial commitment and recognition of contextual factors [14, 23, 24].

The NeoKIP intervention was designed to promote continuous interactions between local stakeholder groups and facilitators and was essentially developed to allow for system integration if found successful. However, little is known with regards to which factors provide for, or hinder, interventions such as these to sustain beyond the lifetime of the particular project. Although this kind of community-based participatory intervention with facilitation as its core strategy is associated with a positive outcome (in this case, reduction in neonatal mortality), a better understanding of the sustainability and potential spread of knowledge implementation is needed–in general and vis-à-vis facilitation in particular [25, 26]. Thus, the aim of this study was to investigate factors which promote or hinder the implementation and sustainability of community-based knowledge implementation strategies, by means of the NeoKIP trial.

## Methods

### Design

This study applied a qualitative design, with focus group discussions (FGDs) and individual interviews across organisational layers of the Vietnamese health system of the former NeoKIP trial area.

### Setting

The NeoKIP trial was undertaken in 90 communes in the Quang Ninh province, located in the northeast of Vietnam. In Quang Ninh there are about 350,000 inhabitants, with 90% belonging to the Kinh majority and the remaining to 10 minority groups (each having an exclusive culture and language). Vietnam is a lower middle-income country [27] with a crude birth rate of 16 live births per 1,000 people, an adjusted maternal mortality ratio of 49 maternal deaths per 100,000 live births and a neonatal mortality rate of 13 neonatal deaths per 1,000 live births (13/1,000) [28] each year. However, there are large variations within the burden of neonatal mortality in Quang Ninh, ranging from <10/1,000, similar to settings in Eastern Europe, to >40/1,000, which is similar to many low-income countries that are associated with pronounced geographic and social inequities [22, 29].

**Fig 1:**
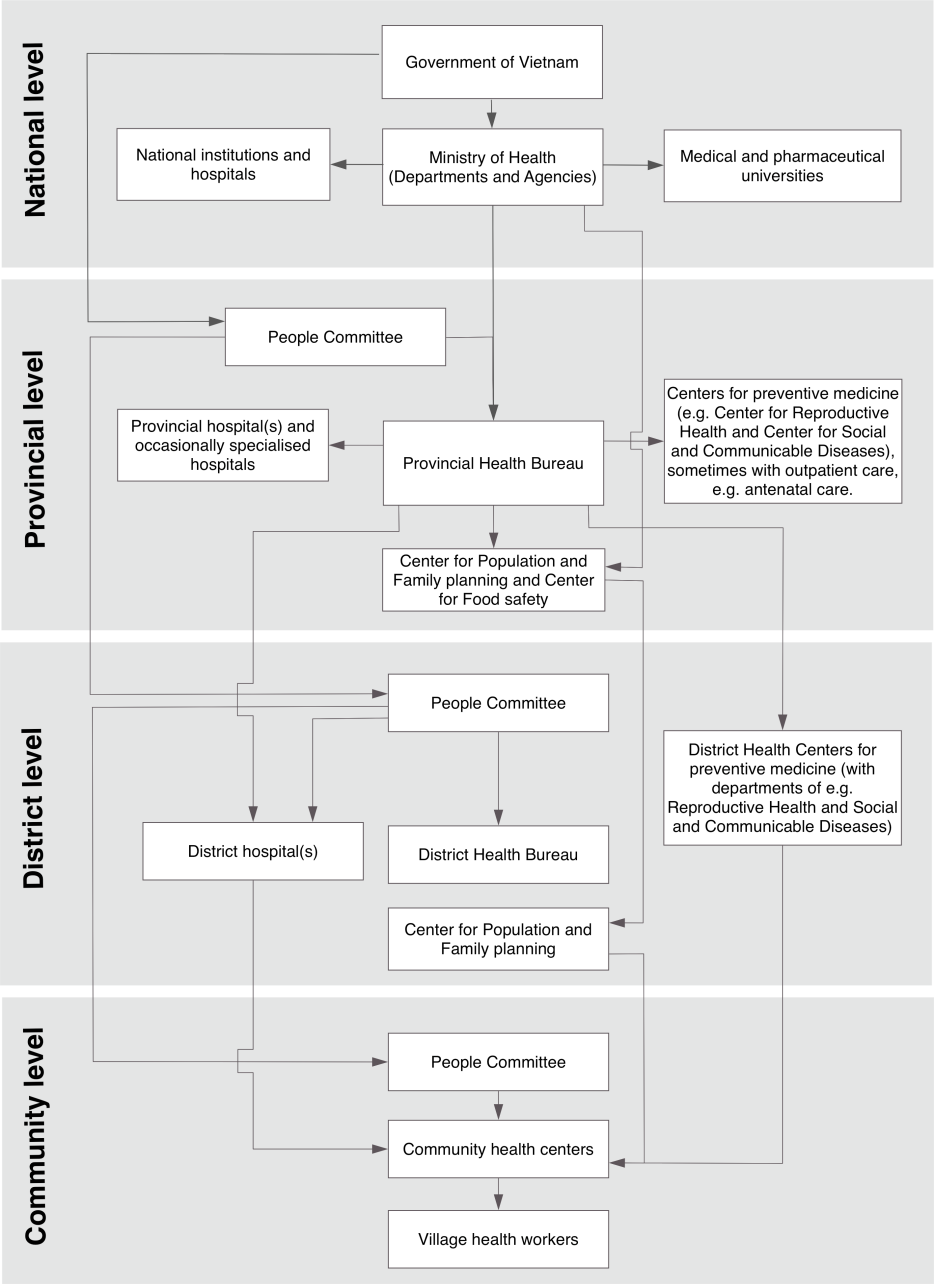
The Vietnamese political/administrative system and healthcare system. Illustration of all levels (national, provincial, district and community) of the Vietnamese political/administrative system and healthcare system.

In the Vietnamese healthcare system, primary healthcare is delivered at commune level, more advanced health care at district and provincial levels, and highly specialised care at national level (Fig 1). Further, parallel to the healthcare system there is a political/administrative system, which consists of parties at provincial, district and community level, controlled by the national government; at each level there is a Peoples’ Council who appoints a Peoples’ Committee with a responsibility to convey and implement government policies. Representatives from the political/administrative system and the healthcare system are supposed to interact on a regular basis at each level (province, district and commune).

### Data collection and analysis

In this study, we purposively targeted representatives of all four levels of the healthcare system (as described above), through individual interviews and FGDs. We conducted six individual interviews (Interview 1–6) at national (*n* = 2), provincial (*n* = 1), and district (*n* = 3) levels, targeting directors of healthcare facilities and representatives of healthcare organizations. This represents all, and is a representative sample at these levels. At commune level, we executed six FGDs (FGD 1–6) with 8–11 individuals in each group who had participated in the former local stakeholder groups in the NeoKIP trial [24], providing for a comprehensive illustration of factors promoting and hindering implementation and sustainability, in general and in NeoKIP. None of the potential participant declined or dropped out of the study.

Separate guides were produced respective to interviews and FGDs (S1 Table). Experienced interviewers who were fluent in Vietnamese and knowledgeable of the NeoKIP trial performed the interviews; all individual interviews were moderated by one researcher with a PhD degree in medicine and the FGDs by another researcher with a PhD degree in sociology. In addition, a public health researcher took notes at all interviews (individual and FGDs). The interviews were performed in Vietnamese in a secured space at the individuals or groups work place and lasted from 45 to 120 minutes. The interviews were audio recorded, transcribed verbatim and then translated into English before analysis. The notes taken were added and marked as additional to the transcribed interviews.

The analysis followed the principles for thematic analysis [30]: initially, all texts were read through several times, providing for a naïve understanding of the whole. Subsequently, the analysis proceeded with an **inductive phase**, when the texts were read repeatedly, and meaning units were identified and labelled with codes. All codes with content directly related to the NeoKIP trial were kept separate, while the analysis proceeded inductively to form sub-themes and, later, themes illustrating features facilitating or hindering knowledge implementation, sustainability of knowledge implementation, and potentials for expansion of knowledge implementation. Further, to complete the analysis, the codes and corresponding meaning units depicting the NeoKIP trial were revisited in a **deductive phase,** where the identified aspects of successful (or unsuccessful) knowledge implementation, sustainability and spread were used as a matrix of the experiences of the NeoKIP trial.

### Ethics approval

To ensure that all participants had the same information, they were verbally informed by the moderator about the study by means of a structured information sheet. In Vietnam, verbal consent to participate in research is standard, and thus more customary than in writing. As a result, all study participants were individually asked about consent to participate before the interviews; the moderator noted the approval of each individual on a separate consent form. The Provincial Department of Science and Technology in Quang Ninh province, Vietnam (ref 3934/QDBYT) and the Research Ethics Committee at Uppsala University, Sweden (ref 2014:205) approved the study.

## Results

The inductive analysis formed two themes (across eight substantiating sub-themes): “Successful projects are agreed, tailored, funded and shared” and “Sustainability in change is nurtured by engaged stakeholders, tailored strategies and sensible use of funds”. An overview of the themes and subthemes is presented in Table 1.

**Table 1:** Subthemes and themes identified in the inductive phase of the analysis.

Subthemes	Themes
The engagement and roles of leaders and participants	Successful projects are agreed, tailored, funded and shared
Projects are monitored	
Projects need tailoring to context	
Management of funding is somewhat flexible but requires transparency	
Engagement, enthusiasm and commitment are important components among leaders and other stakeholders	Sustainability in change is nurtured by engaged stakeholders, tailored strategies and sensible use of funds
The health system’s impact on change	
Tailoring implementation strategy and format	
Funding implementation projects—before, during and after	

### Successful projects are agreed, tailored, funded and shared

#### The engagement and roles of leaders and participants

The interviews depicted that when a project is implemented in the Vietnamese healthcare system (targeting commune or village level) it is commonly managed at provincial level where a project steering committee is established. The project stakeholders, that is, the elected leader and the members of that committee, may vary, depending on the focus of the project. A main task for a project leader is to direct and support project participants at the lower levels. However, project leaders are also in close contact with the higher levels of the healthcare system and sometimes need their approval for moving forward. The project steering committee is mainly active during the initiation phase, but continuously follows up on the project process, whereas project stakeholders and participants are expected to fulfil duties throughout the lifetime of a project.

*“Project stakeholders stay engaged until the end of the project*, *except those who had to retire or were transferred*. *Each stakeholder has a particular responsibility*, *so they have to stay until the end to ensure a complete process*.*”* (Interview 2, District level)

#### Projects are monitored

According to study participants, healthcare projects in Vietnam are, in general, monitored. The monitoring is commonly established in a top-down manner involving many of the health system levels but to a various extent. The monitoring activities can be the responsibility of different project stakeholders, including researchers and funders. Also, regular follow-up is normally established using different methods (e.g. checklists, observations, and interviews), providing feedback to assure the quality of the performance and the fidelity of the planned actions. Sometimes project participants are informed of the planned follow-up visits while, in other cases, the visits are unannounced.

*“By using a checklist or interviewing project participants*, *not only the project leader*, *a supervisor can collect information on how the project activities actually proceed*.*”* (Interview 6, National level)

It was also conveyed that when finishing a project, a final report is produced and the results are disseminated among the relevant stakeholders.

#### Projects need tailoring to context

Informants shared that when setting up a new project it is essential to assess the needs in order to understand the current situation. Also, choosing partners (project stakeholders and participants) who should partake in the project is an important step. These preparations are necessary in order to tailor the project to the setting.

Later, when the results from projects are available, stakeholders have to consider which results to disseminate and how. In the absence of this step, the diffusion of the project’s implementation is jeopardised, however, adopting effective strategies can still promote its sustainability.

*“All successful projects in the community should*, *if possible*, *continue so that people*, *including children*, *can benefit from them and so that our efforts in implementing them are not in vain*.*”* (Interview 1, District level)

#### Management of funding is somewhat flexible but requires transparency

When a project budget is agreed and approved, one share of the budget is usually fixed, while other shares of the budget are more flexible and can be modified based on needs. Transparency of project budgets is deemed important, particularly in relation to sponsors. To establish an effective process, recurrent dialogue is the best approach. If the project steering committee, including the project leader, has a complete overview of the project, they can suggest minor budget adjustments, but approval for the execution of larger changes is commonly needed from higher levels.

*“Usually we have to make requests to higher levels in the healthcare system for adjustments*. *However*, *for those items less than 5 million Vietnamese Dong [≈$250] we can make adjustments ourselves*.*”* (Interview 2, District level)

### Sustainability in change is nurtured by engaged stakeholders, tailored strategies and sensible use of funds

#### Engagement, enthusiasm and commitment are important components among leaders and other stakeholders

A key to sustainability in change is to have well informed leaders, project stakeholders and, not least, community members. When people are aware of why a project is conducted and what benefits it might have, the chances of succeeding are higher. The project leader is in a position where he/she can obtain an overview of the situation, which then allows him/her to ensure that activities comply with guidelines and regulations, and that plans are realistic and appropriate for the situation within a certain context. The project leader is commonly also the person responsible for engaging in a dialogue with other health system levels. In order to sustain project activities, the project leader is thus an important person. If the leader has an interest in a project, this will favor both its implementation and sustainability.

Providing generous information about the project and its benefits will secure the commitment of the committee members and project participants and increase their sense of ownership. However, it is important to reach stakeholders at all levels, that is to say, from those at grass-roots level to the decision makers at higher levels of the healthcare organisation. The characteristics of the project stakeholders are important, as they have influential positions and therefore need to be enthusiastic and have a genuine interest in implementing certain knowledge. While this is something that should already be considered when planning for a project, it was highlighted as being more important in a Vietnamese healthcare context as its people were depicted as being reluctant to join projects.

*“You see*, *when they [potential stakeholders] are asked to do something which differs from their daily routine*, *they tend to reject*, *saying it’s too difficult*. *But once they have engaged in doing it*, *they often find it easy and simple*.*”* (Interview 5, National level)

If the targeted population do not understand the purpose of a project it will be hard to implement change and thus achieve sustainability. However, getting procedures integrated into regular routines can help both the targeted population and the project stakeholders to become more familiar with and actually apply various features of a strategy. To make this happen, a project should have a plan on how to sustain the effect of a beneficial change beyond the project period.

#### The health system’s impact on change

As described above, project stakeholders and project leaders are key in community-based projects. However, participants emphasized that a common problem in the Vietnamese system is the lack of forums and/or time for where and when communication can take place. It was also suggested that poor communication can origin from a lack of interest in certain groups; for example, the district level organisations are not only nodes for information transfer with the provincial and community levels but also between the political/administrative system and the healthcare system. Thus, a lack of engagement at district level is a major barrier for communication.

*“Every month*, *we host a work meeting with CHC heads*. *We have invited representatives from across the district level several times*, *according to the local regulation*. *However*, *they [the District Peoples Committee representatives] fail to attend every time*. *If they would attend the meetings*, *they would get information directly from CHC heads and it would be more adequate and accurate than getting it via us*.*”* (Interview 3, District level)

#### Tailoring implementation strategy and format

To achieve sustainability in the implementation of community-based strategies it was suggested that it is important to, early on in a project, establish strong relations with the “right” people, that is, stakeholders and individuals with authority as well as the recipients of the implementation.

Further, conducting a needs assessment and assessing who to engage early on in the project to ensure that they can have their say prior to the project launch is important. A thorough assessment should consider the current legislation, to assure that the projects are accurately performed. Further, policy advocacy can help a project to succeed; therefore, lobbying for a project before actually getting started may be worthwhile.

*“If a foreigner wants to design a project for the health sector in Vietnam*, *it is important to analyse the needs before setting it up*. *You have to find out what the health sector needs*, *its capacity to meet the requirements*, *what problems it faces*, *and who can help solve such problems*. *… Another factor contributing to ensuring sustainability is dissemination in the community*. *Before the implementation of a project*, *dissemination of the project should be conducted to inform people in the community about it*, *its objectives*, *the approach*, *the benefits it will bring about*, *and what the requirements are for being entitled to benefits*. *This is essential for sustainability*.*”* (Interview 6, National level)

Additionally, if the local authorities need to secure resources in order to sustain a change, for example, to keep people on the payroll once the project funds are withdrawn, it is less appealing than change with no or limited impact on the budget. Further, projects providing resources (e.g. equipment) or resulting in a visible change (e.g. a new building) are more easily accepted than more abstract projects (e.g. to increase knowledge or to empower staff). While the latter type of projects usually encounters more barriers, communities can become committed through the provision of substantial information and communication.

#### Funding implementation projects—before, during and after

There was no common understanding among the informants with regards to the requirement of funding to sustain a project. Rather, some participants described that funding is necessary to sustain change beyond the project period, while others stressed that money is not that important and/or only has short-term effects. To them, including and engaging leaders, project stakeholders and the targeted population working with or being affected by the project is more important. Regardless of this inconsistency, all shared the notion that certain strategies to attain and manage funds are vital for a project, and that the original project plan should include how to hand over once external support ends.

*“To sustain project activities after a project is over*, *we should act in the same way as we adjust medications; doses are gradually cut*. *Thus*, *for project activities*, *funding can be reduced gradually and the project sponsor should carefully consider the cuts*, *and communicate decisions to the head of the local authority*.*”* (Interview 2, District level)

### NeoKIP—An illustration

The deductive phase of the analysis rendered the informants’ experience of the NeoKIP intervention retrospectively. The following aspects were found to support knowledge implementation and sustainability in relation to the former NeoKIP trial: *Engagement*, *Project management*, *Tailoring to context*, and *Funding*.

#### The NeoKIP project–in retrospect

The project not only resulted in a substantial reduction of neonatal mortality but it also had implications for the healthcare system, from both a provider and a user perspective: study participants described that although the regular group meetings at commune level supported by a facilitator ended with the project, the healthcare system, particularly at primary level, focused more on maternal and newborn healthcare after the NeoKIP than before. According to the informants, this was, to some extent, caused by the endurance of NeoKIP: with the project running for three years, healthcare staff became aware that maternal and newborn care is important, and focused more on these aspects in their daily practice. Further, informants suggested that NeoKIP’s focus on maternal and newborn care had affected the population in a positive way; the population was considered to be more aware of different issues related to maternal and newborn healthcare, for example, an increased knowledge of the importance of immunization was noted, leading to increased health service utilization. In particular, NeoKIP was considered to have affected the ethnic minority groups in a positive way.

*“In the past*, *ethnic minority people of my age [around 60] didn’t know at what point they had become pregnant and thus when they would give birth*. *But since the NeoKIP project*, *when all institutions in the commune jointly shared information on this [reproductive health]*, *particularly the younger people have become more knowledgeable*.*”* (FGD 3, Community level)

#### Engagement

The NeoKIP project was depicted as a social intervention where people from various stakeholder groups became engaged in focusing on a common problem area. Communicating with women of reproductive age regarding different matters was considered a particularly key achievement that contributed to the success of NeoKIP. The level of engagement was rather high between group participants and the targeted population. However, the composition of the teams was highlighted as being a barrier. For example, more village health workers should have been included in the groups, and it would also have been appropriate to include other village representatives.

To have facilitators in NeoKIP from the Women’s Union, with the task of supporting people working with maternal and neonatal health, was not entirely appreciated. While the facilitators lacked knowledge in maternal and neonatal care (but were trained in facilitating knowledge implementation) the health professionals described that they wanted more up-to-date knowledge on pregnancy, childbirth, and neonatal care. Also, some facilitators were thought to lack skills in communication, which was considered a key feature in their role. The informants suggested that the group of facilitators in NeoKIP needed more training or to engage healthcare staff from the CHC as facilitators.

*“I believe that the facilitator staff should live and work in the local setting and he or she must have [clinical] expertise to support others*.*”* (FGD 1, Community level)

#### Project management

The success of NeoKIP was partly attributed to its ability to engage various members of the healthcare system, particularly at commune level. However, a further integration of the project into the healthcare system, with the ownership being shared to the administrative and political part of the health system, would have sustained further success and sustainability. Further, increased engagement would also have helped to sustain facilitation of knowledge implementation as proposed in NeoKIP. The project was criticised for lacking the involvement of certain stakeholders, for example, those at district level (district hospitals, district centres and district People’s committees) and for providing opportunities for these parties to interact with project staff.

*“When implementing a project like NeoKIP*, *to have a two-way communication between the health system levels is very important*. *Communes should communicate and report to the district level*, *who can evaluate and give advice*.*”* (Interview 3, District level)

#### Tailoring

NeoKIP was perceived to have been tailored to some extent, for example by including various stakeholders working together with maternal and neonatal health to increase people’s awareness and use of healthcare services. The strategies applied in NeoKIP were still affecting people in that manner after the NeoKIP intervention had ended, and, although no facilitators arranged further meetings beyond the project, the healthcare workers still applied the same approach, focusing on knowledge issues, and employing the means used in the intervention.

*“Although there is no NeoKIP project now*, *the commune still continues its activities and we hold meetings with people from different organizations who interact with the population about perinatal health*.*”* (FGD 4, Community level)

However, NeoKIP was not considered to be sufficiently tailored. For example, the facilitation strategy was deemed to be more suitable for targeting the most remote and difficult-to-reach areas of the province. Also, without a dialogue with the local population prior to the project and with key people at different levels of the healthcare system during the project, sustainability was jeopardised.

#### Funding

Funding during the NeoKIP project was a common issue: with limited reimbursement (provided only as a monthly salary to the facilitators and travel expenses for village representatives to attend monthly group meetings), the informants recalled that there were people who should have been financially compensated during the NeoKIP intervention. Another point raised was the general lack of project funds available, restricting group members to mainly target the areas close by and not those in greatest need. Also, when having meetings at different places in a commune, it would have been appropriate to be able to attract people, which, in the Vietnamese context, necessitates a financial incentive.

*“To tell you the truth*, *it takes them half a day to come all the way here to work for 10*,*000 Vietnamese Dong [about half a US dollar] which is just enough for a drink*. *As we work in the station here*, *it is not an issue for us to attend a meeting*. *But for other members of the board [e*.*g*. *village health workers]*, *it was quite frustrating to do it without being reimbursed*.*”* (FGD 6, Community level)

In addition, informants suggested that for future efforts within NeoKIP it was crucial to have funding for various activities and for supporting participants. When NeoKIP was compared with other projects, those with funding were described as having more satisfied participants. The lack of financial support when ending the project contributed to the threat to its sustainability.

## Discussion

In this study we found factors that are important to consider to achieve the successful implementation and sustainability in change of community-based knowledge implementation strategies. Most importantly, engagement, project management, tailoring and funding play a vital role. This both applies in general in low- and middle-income settings and also, to some extent, illustrates the NeoKIP trial in northern Vietnam.

The aspects identified relate to core components of the integrated Promoting Action on Research Implementation in Health Services (i-PARIHS) framework [15]; i-PARIHS depicts successful knowledge implementation as the interplay between innovation, recipients, context and facilitation. We will discuss what was found to facilitate implementation and sustain endurance of NeoKIP with regards to these factors, and suggest how the findings apply in future implementation efforts in Vietnam and similar settings.

A basis for the NeoKIP project was the excellent evidence available on what types of clinical interventions must be implemented in order to improve neonatal health and survival [2]. In Vietnam, these interventions (corresponding to what i-PARIHS defines as ‘innovation’) were generally available, in the format of national reproductive health guidelines [31]. In addition, various dissemination activities had been performed, for example, in the Quang Ninh province, where information about the clinical interventions had been shared in theoretical and practical training sessions, and via printed materials (e.g. pamphlets and textbooks) [32].

At the launch of the NeoKIP trial, the adoption of the guidelines varied, as did staff’s knowledge about safe procedures for perinatal health [33], but with an almost 50% lower neonatal mortality rate compared to the control communes three years into the NeoKIP study [22]. Even though the health authorities in Quang Ninh decided not to sustain and adopt the NeoKIP strategy when the project ended, the current study shows that the NeoKIP trial’s focus on maternal and neonatal health at commune level still affects current healthcare practice, even without regular meetings with the appointed facilitators. This finding indicates that when recipients identify the benefits of a clinical intervention they are most likely to sustain it in daily practice. Such benefits should be experienced both by the recipients and others [34], in this case, the collaboration on maternal and neonatal health promoted increased standards in quality of care and recognition of this benefit within society.

The i-PARIHS framework suggests that what is referred to as ‘the recipients’ are central to the success of an implementation project; recipients being those *‘who are affected by and influence implementation at both the individual and collective team level’* [15]. In the NeoKIP trial there were both primary recipients (facilitators and intervention group members) and secondary recipients (health system stakeholders and people living in the study area) [22]. There was a great deal of engagement and interaction between the primary recipients and also between primary recipients and family members in the study area, particularly through communication and mobilization activities targeting pregnant women, mothers and other family members [24, 35]. However, in the NeoKIP project the connection with stakeholders at higher levels of the health system rarely occurred, neither before (when planning and initiating the trial) nor during (through regular contact with primary recipients) the trial [21, 24, 35]. Rather, we found that engaging leaders and other key stakeholders is vital to sustain change; although the NeoKIP trial was established in collaboration with representatives from various levels of the health system, those representatives were not sufficiently involved throughout the project’s lifetime. Again, this emphasizes the importance of assessing the needs before initiating a project, that is, by connecting to potential stakeholders and trying to understand which contextual factors might influence implementation already from the beginning [23, 36]. Also, to tailor an intervention to include more health system representatives, particularly those in principal positions, is beneficial for achieving success and sustainability [37]. These findings emphasize the importance of recognising *Co-Creation in Complex systems*, that is, to focus on the various stakeholders in a system and to understand their roles and interaction in a change process [38]. In the current study, it was highlighted that it is important to include representatives from both the political/administrative system and the health system, particularly those having key positions at district level.

Context has been identified as a major influence on knowledge implementation [36, 39]. In the i-PARIHS framework, context depicts both inner and outer context: the inner context represents the local healthcare facility (in the NeoKIP case, primary healthcare) and the organisation where it is embedded [15]. The outer context includes the wider healthcare system, including levels and infrastructures dealing with, for example, policy, regulations and politics. The context construct is closely related to recipients, as they are either active stakeholders in the inner and/or outer context, or are affected by actions taken in either or both levels.

In the current study, participants depicted the importance of including stakeholders from various levels of the healthcare system: this was the road to success for healthcare projects and sustained them over time. However, this implies having a healthcare system susceptible to this kind of interaction. In the Vietnamese system, we identified a number of obstacles to the necessary interactions, for example, key stakeholders in positions of power were not sufficiently engaged in attending important meetings or assisting in sustaining successful projects. Hence, this NeoKIP follow-up reinforces the need to address healthcare systems and cultures across all levels in both the inner and outer context. When recruiting key stakeholders in healthcare organizations their understanding and interest in improvements should be considered [40]. Also, ensuring that there are opportunities for stakeholders from across the healthcare system to interact is vital, prior to, during and after an implementation project [41]. This would enhance the likelihood of success and sustainability of knowledge implementation interventions.

Funding was another important aspect highlighted in regards to accomplishing sustainability in the implementation of community-based strategies. Even with no consensus, if funding is required to sustain change, the study participants agreed on the necessity to have strategies in place for how to manage funds during the project and when handing over the project. This knowledge is of particular importance for health system representatives in countries such as Vietnam; like many other developing countries, Vietnam is in a transition from low-income to a middle-income country [27], with less global attention and external economic support.

Facilitation is suggested as an appropriate implementation strategy in healthcare settings [42]. The i-PARIHS framework defines facilitation as *‘an assigned role to activate implementation by assessing and responding to an innovation and the recipients within a context’* [15]. In this follow-up on the NeoKIP trial, facilitation per se was not perceived as a factor contributing to success in the implementation phase and the strategy did not sustain. However, having engaged and motivated leaders was depicted as important aspects that contributed to the successful implementation and sustaining of various strategies. A facilitator could function as leader in implementation projects, that is, a person who works in an inner context, supporting and interacting with recipients. But a facilitator can also be a person who has acquired an overview of the outer context and has knowledge of how to recruit recipients at various levels. While this was not the case in the NeoKIP intervention, where the facilitators worked within the inner context only [24, 35], it indicates a need for a broader scope for future studies. Further, while interactive skills and communication were criteria for the facilitator role in NeoKIP, additional expertise in the clinical field particular to the intervention should also be considered.

### Strengths and limitations

This study represents participants’ reflections over a period of altogether seven years, including the NeoKIP trial (July 2008 to June 2011) and the post-project period (from July 2011 to June 2014, the latter representing when the interviews were performed). While the data thus represent recalled information [43], the credibility is sustained by the surplus of recollections in terms of details, and because the engagement is mirrored in study participants’ responses and descriptions, along with the variety of study participants, including representatives across the health system (commune, district, provincial and national). This range represents data triangulation [44], in that different sources (informants) contribute to comprehensive understanding of the question in focus, that is, factors promoting or hindering sustainability of community-based knowledge implementation interventions. Further, having multi-lingual collaborators on the data collection and analysis research team allowed for member-checking on interpretation issues. Using the i-PARIHS constructs [15] as a starting point for the discussion was helpful in highlighting and summarising factors to be considered when sustaining community-based interventions.

However, while this study investigates sustainability, defined as the extent to which the NeoKIP intervention kept delivering its intended benefits beyond the implementation period [26]; it focuses the experience of stakeholders involved only. Thus, no data on the sustainability of neonatal health at the follow-up is presented; rather, this study includes what are considered the barriers and facilitators to sustainability in a sample of participants from the former NeoKIP study and additional health system stakeholders engaged in maternal and neonatal health and policy development in Vietnam. As such, the current study provides a qualitative, but not quantitative, perspective on sustaining knowledge implementation interventions in a low- and middle-income context.

## Conclusion

To succeed in implementing and sustaining community-based interventions requires the engagement of leaders and other key stakeholders, the inclusion of all relevant levels in the healthcare system, a thorough needs assessment, a tailoring of the intervention, and a consideration of how to attain and manage funds. Previously, the NeoKIP trial has proved that facilitation by local stakeholder groups is a successful implementation strategy to increase focus on perinatal health and to lower neonatal mortality at commune level. The current study indicates the sustainability of the NeoKIP trial: maternal and neonatal care was still high on the agenda for healthcare workers and leaders long after the trail ended. However, a number of factors attributed to being indicative of success and sustainability were either incomplete or missing in the NeoKIP intervention (such as: thorough assessments prior to implementation of the intervention; tailoring the implementation strategy, and; engagement of key stakeholders). Thus, future launches of NeoKIP and similar knowledge implementation interventions in healthcare have opportunities to adopt a more favourable approach–prior to, during and post trials in low- and middle-income settings.

## Supporting information

S1 Table Interview guides. Interview guides for individual interviews with health system leaders and Focus Group Discussions with former local stakeholder groups.
(DOCX)
