# Supplementary material for: Sustainability of knowledge implementation in a low- and middle- income context: Experiences from a facilitation project in Vietnam targeting maternal and neonatal health
Source: PLoS One. 2017 Aug 14;12(8):e0182626. doi: 10.1371/journal.pone.0182626 (PMC5555694; doi:10.1371/journal.pone.0182626)
Supplement: S1 Table — Interview guides for individual interviews with health system leaders and Focus Group Discussions with former local stakeholder groups. (DOCX) [file pone.0182626.s001.docx]

### Interview guides for individual interviews with health system leaders and Focus Group Discussions with former local stakeholder groups

## Interview guide for individual interviews with health system leaders (Questions in bold and probes within parenthesis)

1. **As far as I understand projects aimed to test the effect of different healthcare interventions are sometimes performed in your area, is that right?** (Could you tell me about some of these recent projects? Any particular example of a project with positive effects? Who initiated/supported these projects?)
2. **If you think about these kinds of projects - what is usually your role in these projects?** (Who is usually involved in the initial phase of new projects and what is your role? Are the same people involved during initiation also involved when the project is running? When these project are running what is your role and how is a person in your position informed about the progress/process? How are findings from these projects communicated to you? (if “they’re not” then pursue asking about what happens)? Who else would be involved, besides you, as a decision maker?)
3. **What usually happens when these projects end?** (What is your role in deciding what happens? What influences what happens?)
4. **Provincial/District level: When it comes to start using a strategy (in your province/district) that has been proven successful in a project – can you tell me what happens?** (Who is usually involved? What is your role in this process? Do you have certain procedures (ways of working) to follow to assure a successful implementation? If not, what normally happens?)

**National level: When it comes to scale-up a strategy found successful in one province to other parts of the country – can you tell me how that is done?** (Who is usually involved? What is your role in this process? Do you have certain procedures (ways of working) to follow to assure a successful implementation? If not, what normally happens?)

1. **How would you describe the flexibility in the budget you have in terms of allowing you to invest in/taking up and implementing initiatives you identify as beneficial for the population you serve?** (If having budget for such initiatives, on what basis do you decide how to use this budget? How come certain things become focused but not others?)
2. **Between 2008 and 2011 there was a project called NeoKIP that was running in Quang Ninh province. In this project half of the included communes had facilitators recruited from the Women’s Union supporting local stakeholder groups. The groups included both primary healthcare staff and local key persons. Do you recall this project** (Question at District and Provincial level) **/ Have you ever heard of this project** (Question at National level)? **In any case, what the teams did was that they identified local problems impacting on neonatal health and survival and developed local strategies to overcome these problems. The project was running with a low budget and only travel expenses were reimbursed. When the project ended the researchers found that in the communes having a group supported by a facilitator, the neonatal mortality rate was 50% lower during the third year than in the communes without such a group. Regardless of whether you remember the particular project or not, why do you think it succeeded?**
3. **It seems that the groups have not continued after the study was completed. We are very keen to understand why – do you have any ideas?** (If I were to ask you, what would you have done, or did you do, to sustain the teams and the strategy they applied? Although the intervention of having groups meeting to solve local problem has not continued, do you think the project might have influenced/initiated anything else that has sustained?
4. **In this discussion with you I have been trying to understand what factors are important when implementing successful strategies/interventions aiming to improve healthcare quality and patient outcomes. Is there anything in particular that comes to your mind regarding this?** (If not now, please don’t hesitate to contact me by phone or e-mail with anything that may refer to what we have talked about – big or small things are just as interesting to us.
5. **We are very grateful for the time you have spent talking to me and for helping us understand more of how the NeoKIP turned out. Thank you!**

## Interview guide for Focus Group Discussions with former local stakeholder groups (Questions in bold and probes within parenthesis)

1. **We met more than three years ago (at the end of the NeoKIP intervention). Do you remember? Tell me; how have you been?** (Warming-up question where the moderator need to let the participants to have time to express their thoughts)
2. **When you think back of this time, tell me, what has happened since then?** (When the regular meetings within the NeoKIP project ended, did you proceed to have meetings, what happened, any other collaboration with those who used to be members of the local stakeholder group?)
3. **During these three years (after the intervention) have any changes occurred in your commune? (**Aspects of the social, political, and economic environment; Changes in relation to the community health centre and/or the healthcare system)
4. **As you may have heard, when we analysed the results from the NeoKIP project we found that in the communes having a local stakeholder group like yours, the neonatal mortality rate was 50% lower in the third year than in the communes without a group. What do you think of this?** (Why do you think this happened?)
5. **In NeoKIP, we focused on neonatal health. Have neonatal health also been on the agenda after NeoKIP?** (If not, why? If yes: how, please exemplify)
6. **The NeoKIP model implied joining several organizations (Community health centre, the commune, and the Women’s unions) in collaboration. Has that influenced how you work/collaborate today?** (If yes: how? If not: why not?) **What could have helped you to adapt to this model to a greater extent?**

1. **If you look at it from your position as a professional (e.g. Chairman, Women’s union worker, primary healthcare worker or village health worker) - do you think that you (as an individual) have changed your way of working in any way?** (How? Compared to the situation before, during and/or after NeoKIP)
2. **What activities/processes from NeoKIP would you liked to continue with?** (Having monthly meetings with the facilitator, collaborate with others about neonatal health, etc) **Tell me, what was needed for this to continue?** (From community, health system or social system?)
3. **Thinking back of NeoKIP – what’s your best memory?** (Closing question)
